# Profiling the Bisecting *N*-acetylglucosamine Modification in Amniotic Membrane via Mass Spectrometry

**DOI:** 10.1016/j.gpb.2021.09.010

**Published:** 2022-02-03

**Authors:** Qiushi Chen, Yuanliang Zhang, Keren Zhang, Jie Liu, Huozhen Pan, Xinran Wang, Siqi Li, Dandan Hu, Zhilong Lin, Yun Zhao, Guixue Hou, Feng Guan, Hong Li, Siqi Liu, Yan Ren

**Affiliations:** 1 BGI-Shenzhen, Shenzhen 518083, China; 2 Joint International Research Laboratory of Glycobiology and Medical Chemistry, College of Life Sciences, Northwest University, Xi’an 710069, China; 3 Shenzhen Seventh People's Hospital, Shenzhen 518081, China; 4 Institute of Interdisciplinary Integrative Medicine Research, Shanghai University of Traditional Chinese Medicine, Shanghai 201203, China

**Keywords:** Glycan, Glycoprotein, Amniotic membrane, Bisecting *N*-acetylglucosamine, Mass spectrometry

## Abstract

**Bisecting *N*-acetylglucosamine** (GlcNAc), a GlcNAc linked to the core β-mannose residue via a β1,4 linkage, is a special type of *N*-glycosylation that has been reported to be involved in various biological processes, such as cell adhesion and fetal development. This *N*-glycan structure is abundant in human trophoblasts, which is postulated to be resistant to natural killer cell-mediated cytotoxicity, enabling a mother to nourish a fetus without rejection. In this study, we hypothesized that the human **amniotic membrane**, which serves as the last barrier for the fetus, may also express bisected-type **glycans**. To test this hypothesis, glycomic analysis of the human amniotic membrane was performed, and bisected *N*-glycans were detected. Furthermore, our proteomic data, which have been previously employed to explore human missing proteins, were analyzed and the presence of bisecting GlcNAc-modified peptides was confirmed. A total of 41 **glycoproteins** with 43 glycopeptides were found to possess a bisecting GlcNAc, and 25 of these glycoproteins were reported to exhibit this type of modification for the first time. These results provide insights into the potential roles of bisecting GlcNAc modification in the human amniotic membrane, and can be beneficial to functional studies on glycoproteins with bisecting GlcNAc modifications and functional studies on immune suppression in human placenta.

## Introduction

A distinctive structural feature of N-glycans is the presence of several N-acetylglucosamine (GlcNAc) antennae that are sequentially synthesized by a group of Golgi-resident glycosyltransferases called N-acetylglucosaminyltransferases (GlcNAc-Ts) [1], [2]. There are three categories of N-glycans: high-mannose, hybrid, and complex N-glycans. Hybrid and complex N-glycans may carry a bisecting GlcNAc group, which forms a new subtype of glycan called bisected glycans [3], [4]. This type of glycan was reported in the 1970s and was detected using a combination of sequential exoglycosidase digestion, methylation derivatization, acetolysis, and Smith degradation of ovalbumin [5], [6]. GlcNAc is transferred to the 4th-position of the β-linked core mannose (Man) residue in complex or hybrid N-glycans by β1,4-mannosyl-glycoprotein 4-β-N-acetylglucosaminyltransferase (GlcNAc-T III); however, this GlcNAc is usually not considered as an antenna initiation point, as it cannot be further extended [7], [8].

GlcNAc-T III is encoded by the gene, *mgat3*, which was initially discovered in hen oviducts in 1982 [9]. The presence of a bisecting GlcNAc prevents α-mannosidase II from trimming and has been reported to inhibit the activities of GlcNAc-T II, GlcNAc-T IV, and GlcNAc-T V *in vitro* [2], [10]. Bisecting GlcNAc is essential for many biological processes, including tumor development and immune responses [11], [12]. For instance, human K562 cells are easily killed by natural killer (NK) cells; however, after transfection with the *mgat3* gene, K562 cells become resistant to NK cells owing to the expression of bisecting GlcNAc [13], [14].

In the 1990s, Clark et al. proposed the human fetoembryonic defense system (hu-FEDS) hypothesis to justify how a mother nourishes a fetus within her body for several months without rejection [15], [16]. This question was initially raised by Sir Peter Brian Medawar, who shared the 1960 Nobel Prize in Physiology or Medicine with Sir Frank Macfarlane Burnet. Since the proposal of the hypothesis, it has been tested intensively, with increasing evidence supporting it [16], [17]. In 2016, Clark et al. found that the functional glycan structure (bisecting GlcNAc) on human gametes is also expressed in human trophoblasts, and more importantly, most N-glycans in human trophoblasts possess a bisecting GlcNAc [7], [18]. Klisch et al. found that bisecting GlcNAc appeared to be a general feature in binucleate trophoblast cells in the ruminant placenta; however, how the bisecting glycan is involved in normal pregnancy and spontaneous miscarriage was not clearly illustrated [19], [20]. GlcNAc-T III is localized within the cytotrophoblast, syncytiotrophoblast, trophoblast columns, and some extravillous cells in the maternal decidua; however, the silencing of GlcNAc-T III, which results in the blockade of bisecting GlcNAc, leads to the inhibition of a series of events, such as invasion and migration of HTR8/SVneo cells and outgrowth of extravillous explants. Extensive efforts are still warranted to understand the involvement of bisecting GlcNAc in pregnancy [21], [22]. The occurrence of N-glycans with a bisecting GlcNAc modification on glycoproteins has been reported to have many implications in immune biology [23], [24].

The amnion is the innermost layer of the placenta and is the last barrier for the fetus. The amniotic membrane is also considered a potential stem cell reservoir with wide applications in periodontics, tissue regeneration, and surgery [25], [26]. Gaining more knowledge about the amniotic membrane is thus becoming increasingly worthwhile. In fact, the amniotic membrane is an optimal sample for analysis, as the possibility of contamination by blood cells, neurocytes, and lymphocytes is low if the membrane is washed in saline or phosphate buffered saline (PBS) prior to enzyme digestion [27]. More importantly, glycomic and glycoproteomic studies of the amnion have not been reported. In this study, we hypothesized that the human amniotic membrane may also express bisected-type glycans. To test this hypothesis, we employed a mass spectrometry (MS)-based approach to determine the presence of bisecting GlcNAc on amniotic membrane proteins. In addition, the corresponding bisecting GlcNAc-modified glycoproteins were determined.

Various MS-based approaches have been developed and proven to be efficient tools for bisecting GlcNAc modification studies, as reported in our recently published review [28]. The basic idea of the glycan method is to determine the presence of 3, 4, 6-linked Man, which can be achieved via either MS7 analysis [29] or partially methylated alditol acetate (PMAA) derivatization with gas chromatography–mass spectrometry (GC–MS) [18]. The basic idea of the glycopeptide method is to determine the presence of the characteristic fragment ion(s) [Peptide + N-acetylhexosamine_3_hexose (HexNAc_3_Hex)] or [Peptide + fucoseN-acetylhexosamine_3_hexose (FucHexNAc_3_Hex)], alone or in combination, which can be achieved through MS2 fragmentation under low-energy collisions, as described previously [30], [31].

## Results and discussion

### Determination of human amniotic membrane protein concentration

**Supplementary figure S1:**
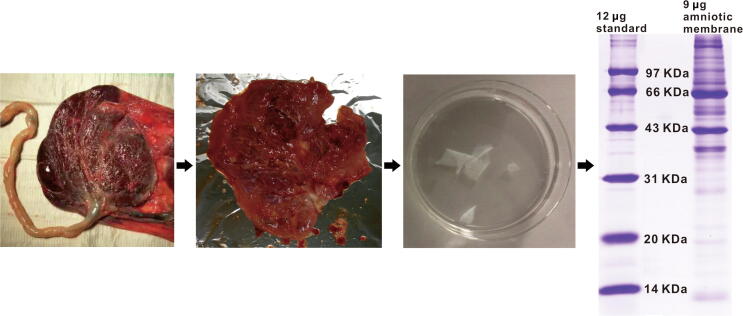
**Human placenta processing**. **A**. Human placenta and umbilical cord. **B**. chorion and amniotic membrane section. **C**. amniotic membrane after washing with PBS. **D**. SDS–PAGE image of the extracted proteins from human amniotic membrane. PBS, phosphate buffered saline; SDS–PAGE, sodium dodecyl sulfate polyacrylamide gel electrophoresis.

The human amniotic membrane was isolated from the placenta (Figure S1A–C) [27]. Thereafter, proteins were extracted using 8 M urea as reported previously [32]. The protein concentrations in homogenized amniotic membranes were determined using a Bradford assay, and the rationality of the protein concentration was determined via sodium dodecyl sulfate polyacrylamide gel electrophoresis (SDS–PAGE) (Figure S1D).

### MS analysis of the amniotic membrane

**Figure 1:**
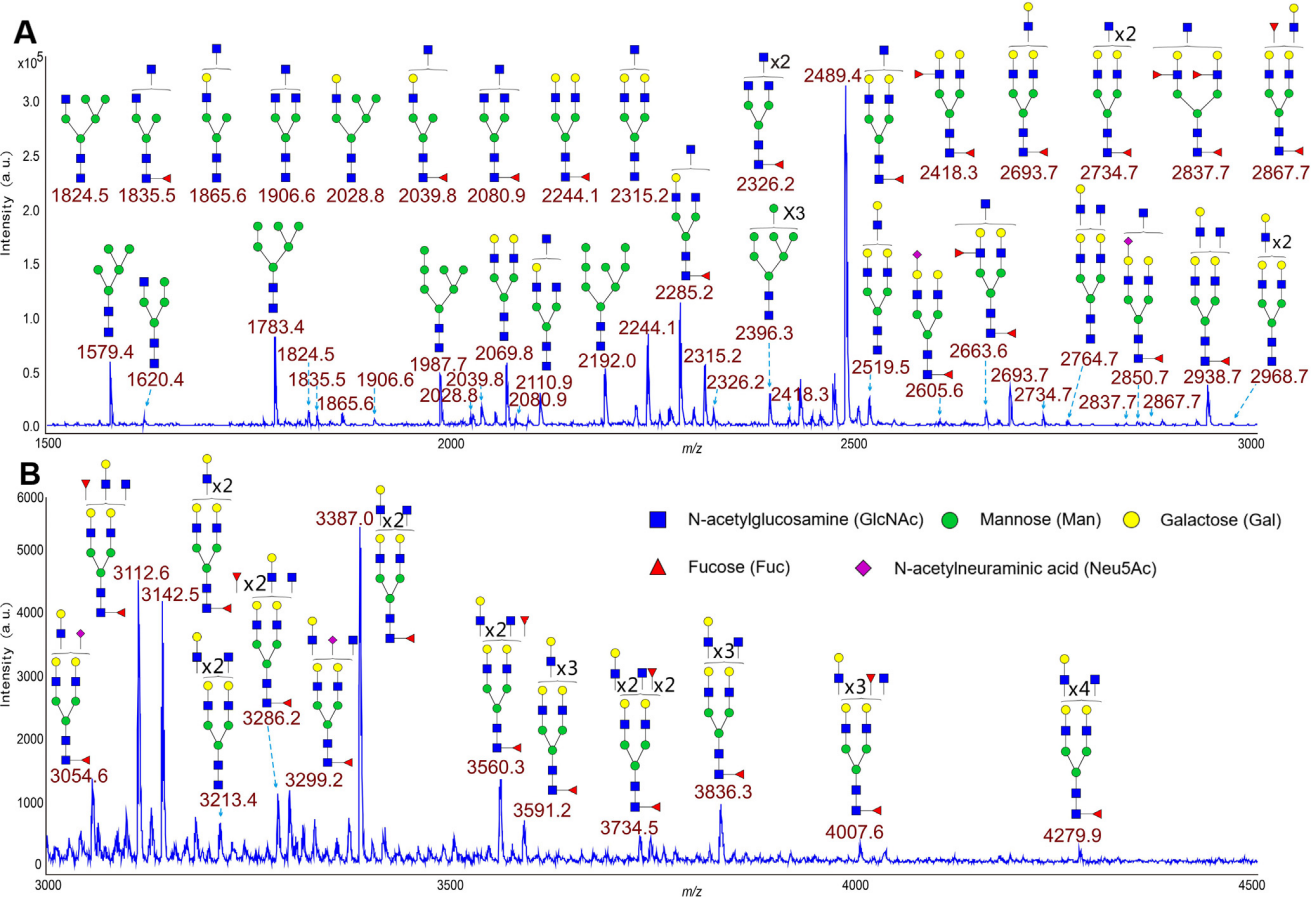
**Annotated MALDI**–**TOF MS spectra of permethylated N-glycans from the human amniotic membrane** The spectra have been smoothened and the baselines have been subtracted with flexAnalysis default setting. **A.** The glycans in the mass range from *m/z* 1500 to *m/z* 3000. **B.** The glycans in the mass range from *m/z* 3000 to *m/z* 4500. All ions are [M+Na]^+^. Peaks are labeled with their *m/z* values, and putative structures are described based on the molecular weight and N-glycan biosynthetic pathway. Annotations are simplified to biantennary structures, with additional LacNAc, Fuc, and GlcNAc listed outside the bracket. MALDI–TOF MS, matrix-assisted laser desorption ionization time of flight mass spectrometry; LacNAc, N-acetyllactosamine; Fuc, fucose; GlcNAc, N-acetylglucosamine; Man, mannose; Gal, galactose; NeuAc, N-acetylneuraminic acid.

The N-linked glycans were released from the amniotic membrane proteins and permethylated for matrix-assisted laser desorption ionization time of flight (MALDI–TOF) analysis. Thereafter, these glycans were subjected to glycomic profiling analysis, as described previously [33], [34]. High-quality MALDI–TOF MS data were obtained for these N-glycans, and a total of 45 N-glycans were identified (Figure 1). High mannose (*e.g.*, *m/z* 1579.4 and *m/z* 1783.4) and complex glycans (*e.g.*, *m/z* 2489.4 and *m/z* 2938.7) were identified in the human amniotic membrane. Several common features of mammalian cell N-glycans have been observed, such as core fucosylated GlcNAc (*e.g.*, *m/z* 2693.7), N-acetylneuraminic acid (NeuAc) capped antennae (*e.g.*, *m/z* 2605.6) and N-acetyllactosamine (LacNAc) units (*e.g.*, *m/z* 3591.2), which form tandem repeats to yield oligo-LacNAc antennae in some cases [35], [36]. The complex glycans accounted for nearly 74% of all detected glycans, and approximately 80% of complex-type glycans carried core α1–6 linked fucose. The *m/z* value of the most complex N-glycan was *m/z* 4279.9, and it contained 19 monosaccharides. Importantly, the peak at *m/z* 2489.4 was found to be the most abundant and was speculated to be a potential core-fucosylated biantennary bisected glycan structure. The sialylation level of glycoproteins from the human amniotic membrane was remarkably lower than that in the human colon, heart, and kidney (Figure S2).

### Verification of the presence of bisecting GlcNAc

**Figure 2:**
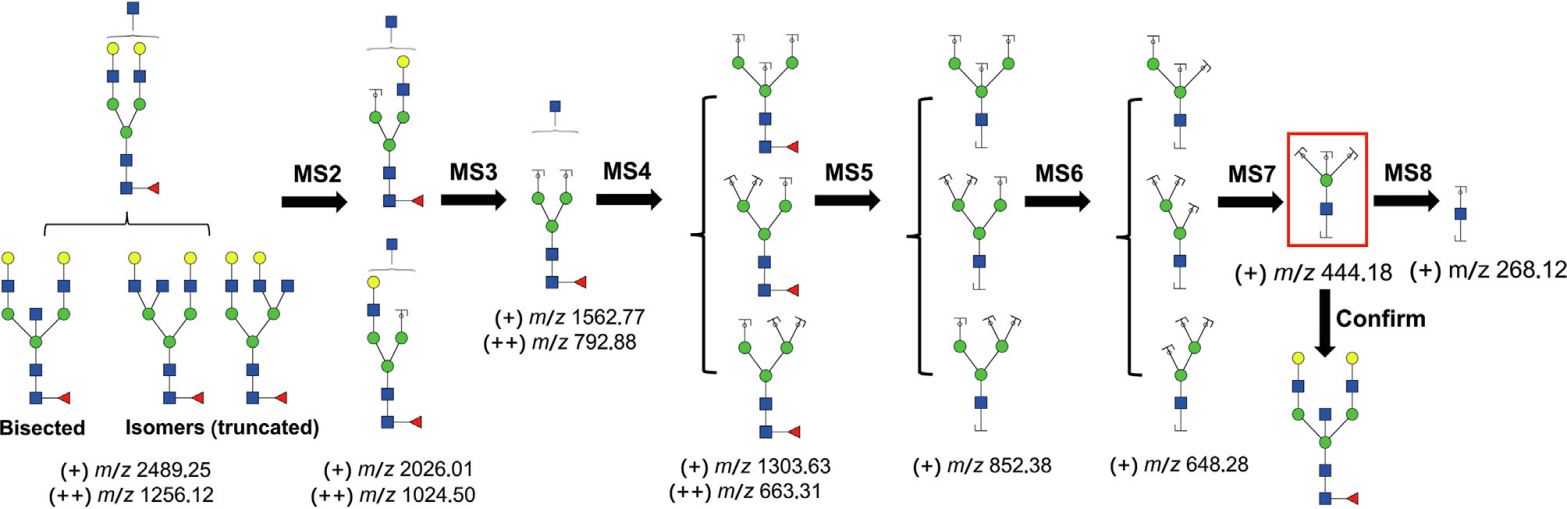
**The MS8 approach confirms the presence of bisecting GlcNAc** The fragment ion at *m/z* 444.18 (theoretical value) in the red frame is the characteristic ion of the bisected-type glycan. MS, mass spectrometry.

The query glycans were further fragmented using the multi-stage MS (MS^n^) (*n* = 2–8) analysis mode to obtain their fine structures. The sequential collision of the target glycan at *m/z* 2489.4 (theoretical *m/z* 2489.25) was carried out to confirm the presence of bisecting GlcNAc. Figure 2 shows the logical order of the MS8 approach, which can determine the presence of a bisecting GlcNAc structure in a glycan at *m/z* 2489.25.

**Figure 3:**
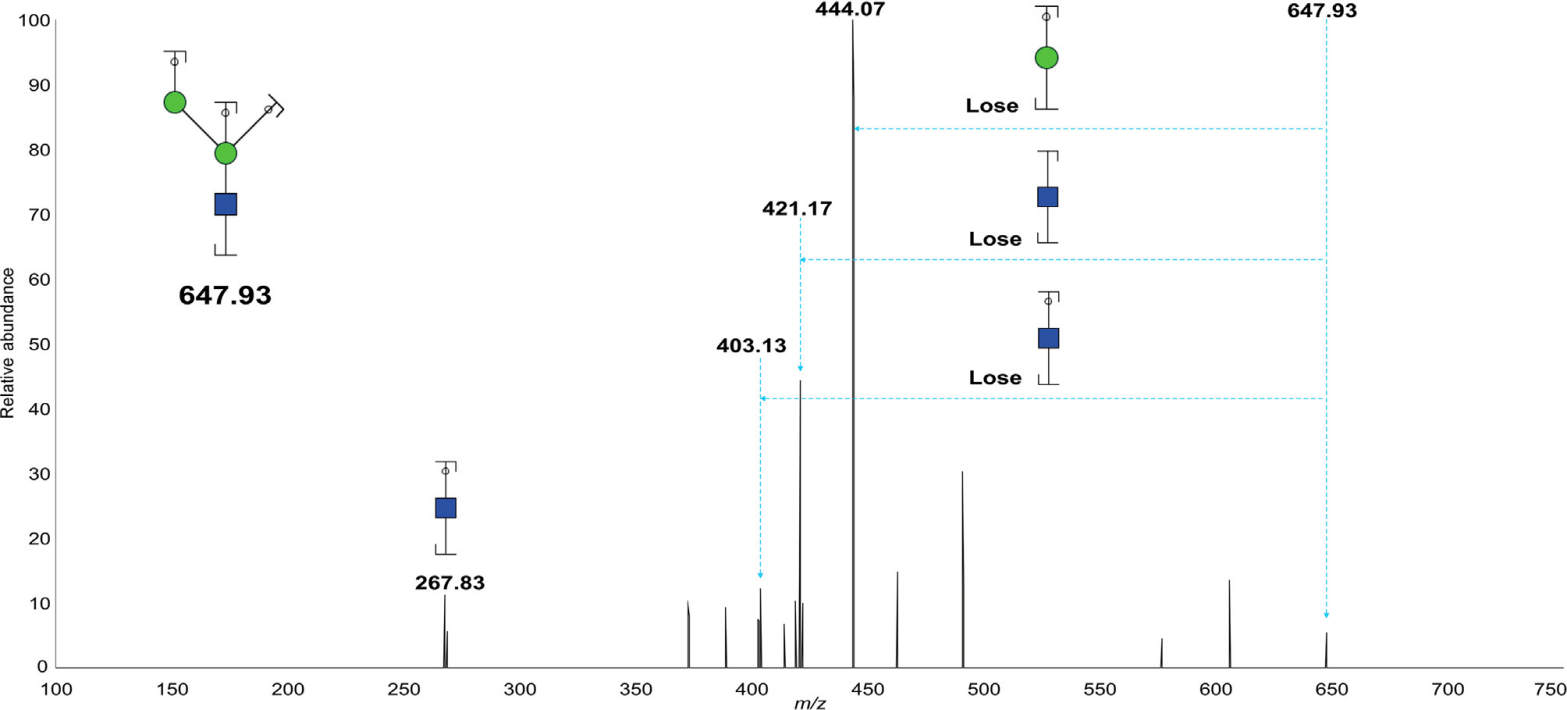
**Annotated ESI ITMS MS7 spectrum of thepermethylated N-glycanion at *m/z*2489.25from the human amniotic membrane** Assignments of the possible fragment ions are indicated on the cartoons and on the spectrum. The number indicated above the peak is the *m/z* value of the fragment ion (resulting ion) that has been detected by the mass spectrometer. Data were acquired in the form of [M+Na]^+^. The energy under CID and HCD modes first breaks glycosidic bonds to form ‘b’ ions or ‘y’ ions, and further fragmentation will produce ‘by’ ions or ‘yy’ ions or ‘bb’ ions, and so on. ESI, electrospray ionization; ITMS, ion trap mass spectroscopy; CID, collision-induced dissociation; HCD, high-energy collision dissociation.

According to the logical order shown in Figure 2, the fragmentation of the *m/z* 648.28 ion is the key step for confirming the bisected structure. Theoretically, after a series fragmentation of glycan at *m/z* 2489.25, the presence of bisected glycan should be supported by the characteristic ions at *m/z* 444.18 in the MS7 spectrum. In fact, the MS7 spectrum (Figure 3) was found to contain a dominant fragment ion at *m/z* 444.07, which differs by approximately 0.11 Dalton from its theoretical *m/z* value. Because of the low resolution of ion trap mass spectroscopy (ITMS), the ion *m/z* values had a relatively high mass error, which differs from the MS1 and MS2 analyses performed via Fourier transform mass spectrometry (FTMS) [37], [38]. As shown in Figure 3, the characteristic fragment ion at *m/z* 444.07 was produced by *m/z* 647.93 (theoretical *m/z*: 648.28) via the loss of Man as a ‘by’ ion (the green circle). In addition to this ion, three ions, *m/z* 421.17, *m/z* 403.13, and *m/z* 267.83 were observed. The ion *m/z* 421.17 is from *m/z* 647.93 via losing a GlcNAc ‘bz’ ion, and *m/z* 403.13 is also a resulting ion from *m/z* 647.93. The ion *m/z* 267.83 represents a GlcNAc ‘by’ ion. Additionally, MS8 analysis was performed to demonstrate that the ion at *m/z* 444.07 is indeed a glycan fragment ion and not noise. All other MS^n^ (*n* = 2–6, 8) spectra of the glycan are shown in Figure S3 (the spectrum of MS^n^, *n* = 7 is shown in Figure 3). Based on all of the fragmentation information, the structure of bisected N-glycans was clarified based on solid evidence of sequential fragmentation using mass spectrometry.

Compared to other MS methods for detecting bisecting GlcNAc modification, our method does not require extra enzymatic treatments (*e.g.*, β1,4-galactosyltransferase) or extra derivatization procedures (*e.g.*, partially methylated alditol acetate derivatization) or lectin recognition (*e.g.*, *Phaseolus vulgaris* erythroagglutinin), which makes this method a straightforward approach for bisecting GlcNAc determination [7], [39], [40]. Such approach could thus markedly facilitate research on bisected N-glycans.

### Fourty one glycoproteins in human amniotic membrane with the bisecting GlcNAc structure

As the presence of bisecting GlcNAc modification in the human amniotic membrane has been confirmed, it is necessary to determine the origin or location of this type of glycan modification. We published an extensive proteomic analysis of human amniotic membranes, where peptides were fractionated using a three-dimensional separation approach according to their hydrophobicity. Thus, the data for 40 peptide fractions with each injection performed for a 2-h MS run were collected and stored in the public database (ProteomeXchange: PXD010630). The data were originally obtained from our previous publication in 2018 [41]. The deep analysis resulted in 9941 proteins with 163,091 peptides detected in human amniotic membranes. Glycopeptides from the library were searched using a combination of Proteome Discoverer, Byonic, and Xcalibur software to determine the glycopeptides and manually check oxonium ions, monosaccharides, and oligosaccharide neutral loss patterns in MS2 spectra to obtain the corresponding glycan structure.

With confident analysis that one glycopeptide was supported by at least two mass spectra, 43 glycopeptides belonging to 41 glycoproteins were found to possess bisected N-glycans, in which the glycosylation sites and linked glycans were confirmed by manual checking in the presence of either [Peptide + HexNAc_3_Hex] or [Peptide + FucHexNAc_3_Hex] or both. Table S1 summarizes all glycoproteins with bisected N-glycan modifications, including their accession numbers, protein names, peptides modified by bisecting GlcNAc glycans, and cellular locations. All glycoproteins were assigned as membrane proteins or extracellular matrix proteins via search of the UniProt library, which indicated that 18 and 22 proteins belong to the cell membrane and extracellular matrix proteins, respectively, and only one protein is derived from the Golgi apparatus membrane. Of all the glycoproteins, approximately 39% have been reported as proteins with bisected N-glycans, such as tumor necrosis factor receptor superfamily member 11B [29], laminin subunit alpha-5 [39], [42], decorin [30], lysosome-associated membrane glycoprotein 2 [30], neprilysin [30], thy-1 membrane glycoprotein [29], nidogen-2 [30], integrin beta-1 [43], [44], cell surface glycoprotein MUC18 [29], [39], transferrin receptor protein 1 [44], integrin alpha-V [44], laminin subunit beta-2 [39], [42], tyrosine-protein kinase receptor UFO [39], immunoglobulin alpha-2 heavy chain [45], [46], carcinoembryonic antigen-related cell adhesion molecule 1 [39], and fibronectin [39]. The other 25 glycoproteins were recognized for the first time as proteins possessing bisecting GlcNAc with reliable MS2 evidence.

**Figure 4:**
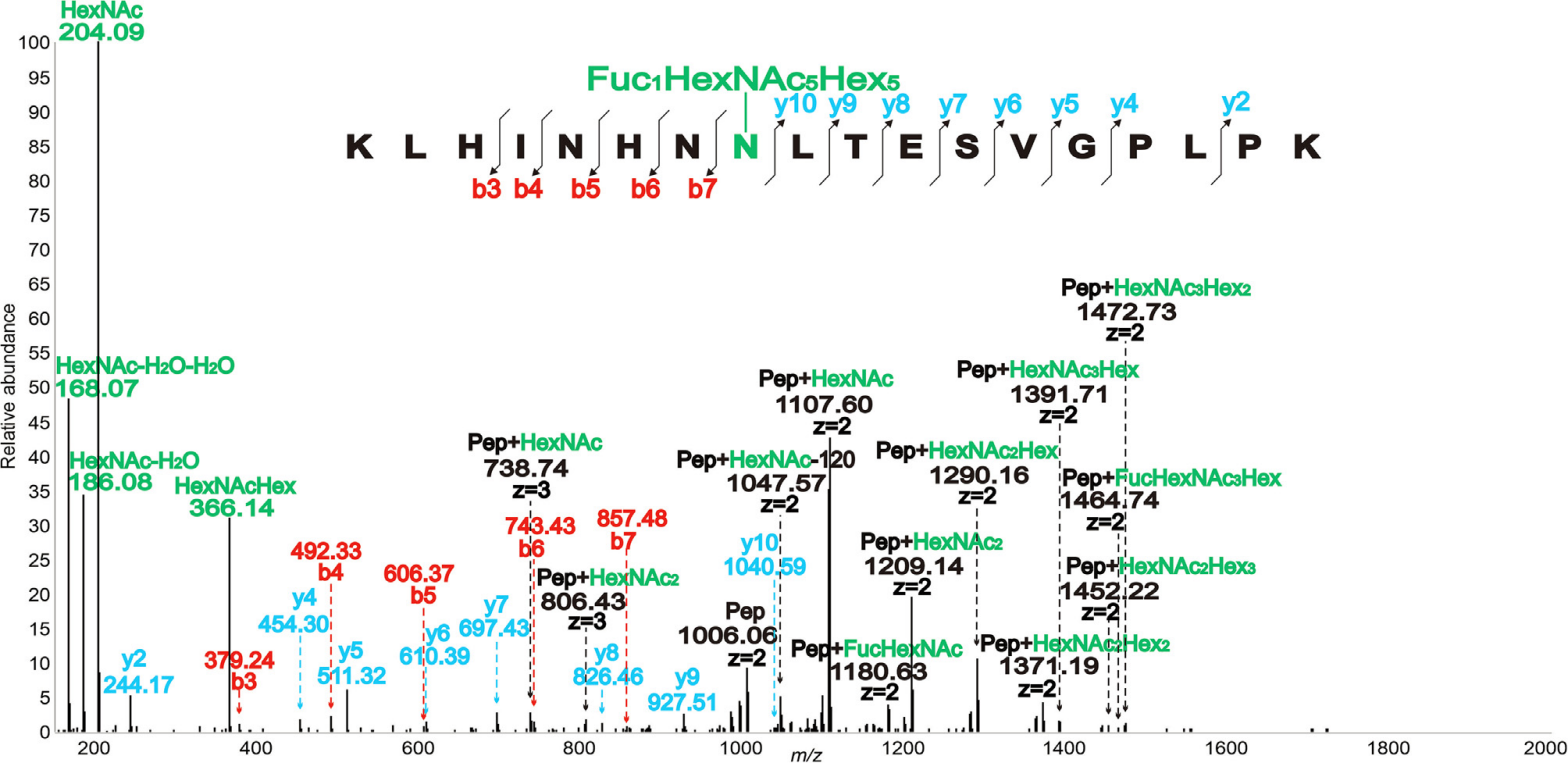
**Annotated ESI MS2 spectrum showing N-glycosylation on the N8 in the peptide KLHINHNNLTESVGPLPK** This Pep is from lumican (UniProt: P51884). All ions are [P + H]^+^ or [P + 2H]^2+^ or [P + 3H]^3+^. Double charged ions are annotated as *z* = 2, triple charged ions are annotated as *z* = 3, and others are monocharged. The number indicated above the peak in the spectrum is the *m/z* value of the ion that has been detected by the mass spectrometer. To make the annotation clearer, ions are labeled in different colors. Pep, peptide.

Figure 4 displays a typical MS2 spectrum annotated as the glycopeptide KLHINHNNLTESVGPLPK, in which N8 (asparagine) in NLT is glycosylated by a bisecting GlcNAc structure. The characteristic fragment ions that usually mark glycosylation presence, such as *m/z* 204.09 and *m/z* 366.14, were clearly observed in the spectrum due to their higher intensity. Further, its 5 peptide ‘b’ ions (b3, b4, b5, b6, and b7) and 8 peptide ‘y’ ions (y2, y4, y5, y6, y7, y8, y9, and y10) support peptide sequence identification and the potential glycosylation modification location. The peaks at *m/z* 1391.71 (*z* = 2) and *m/z* 1464.74 (*z* = 2) matched the [Peptide + HexNAc_3_Hex] and [Peptide + FucHexNAc_3_Hex] ions of the glycopeptide, thereby further proving that the glycosylation modification occurs on N8 and contains a special bisecting GlcNAc structure, according to the ions specifically yielded by bisecting GlcNAc glycans. This glycopeptide is from lumican (UniProt: P51884), and several studies have reported its vital roles in regulating tissue repair and embryonic development [47], [48].

The method employed for bisecting GlcNAc determination was performed as reported previously [30], which was designed to determine bisecting GlcNAc on glycopeptides by their characteristic ion(s) in fragmented MS2 spectra under low-energy collision. Dang et al. identified 25 glycoproteins (possessing bisecting GlcNAc) in rat kidney tissue [30], 4 of which (UniProt: Q01129, P17046, P07861, and B5DFC9) were found to be protein analogs of those identified in our human amnion sample. More importantly, rat neprilysin had the same bisecting GlcNAc location (site N285) as human neprilysin (UniProt: P08473) in our amnion sample.

The same strategy was thus adopted to search for glycopeptides with bisecting GlcNAc structures against the proteomic data collected from the human bladder, kidney, and stomach, which was reported in our previous publication [49]. No bisecting GlcNAc structure was found in the corresponding peptides from human bladder and kidney proteins. However, in the human stomach, there was one glycopeptide, HYTNSSQDVTVPC(carbamidomethyl)R from the human immunoglobulin alpha-2 heavy chain (UniProt: P0DOX2), in which N-glycosylation occurred on the N in the NSS, and its glycan comprised of FucHexNAc_5_Hex_5_. Such finding suggests that some bisecting GlcNAc modifications might specifically occur in the proteins expressed in the human amniotic membrane, which are essential for embryo development.

### Predicted functions of the glycoproteins with bisecting GlcNAc modification

**Supplementary figure S4:**
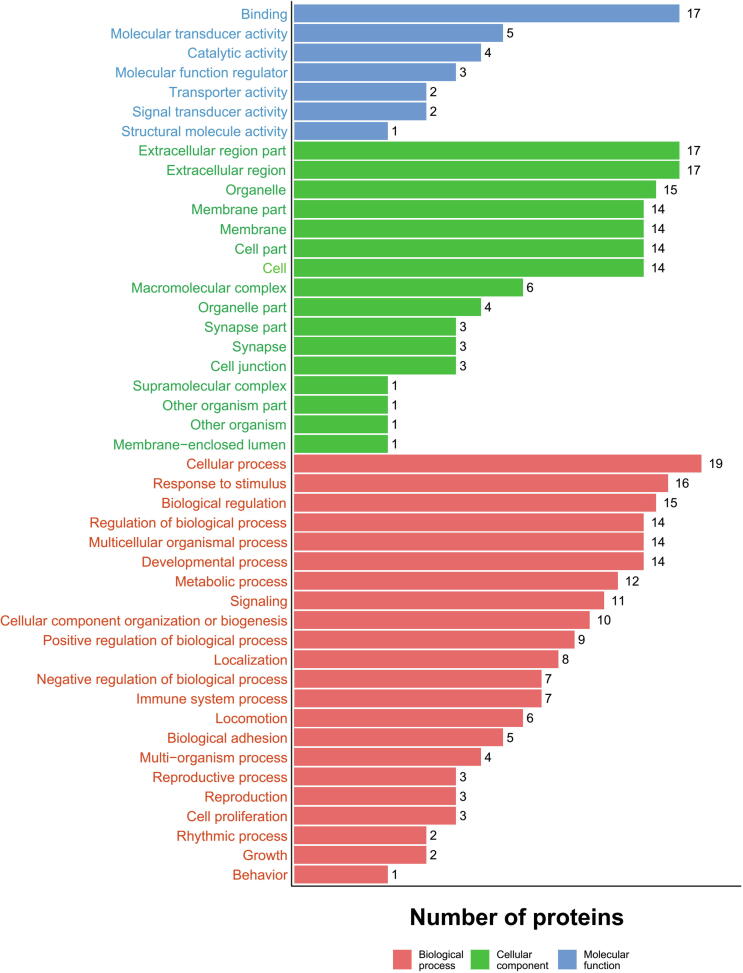
**Distribution of identified bisecting GlcNAc containing glycoproteins in terms of molecular function, cellular component, and biological process**. One protein may be involved in multiple categories.

**Supplementary figure S5:**
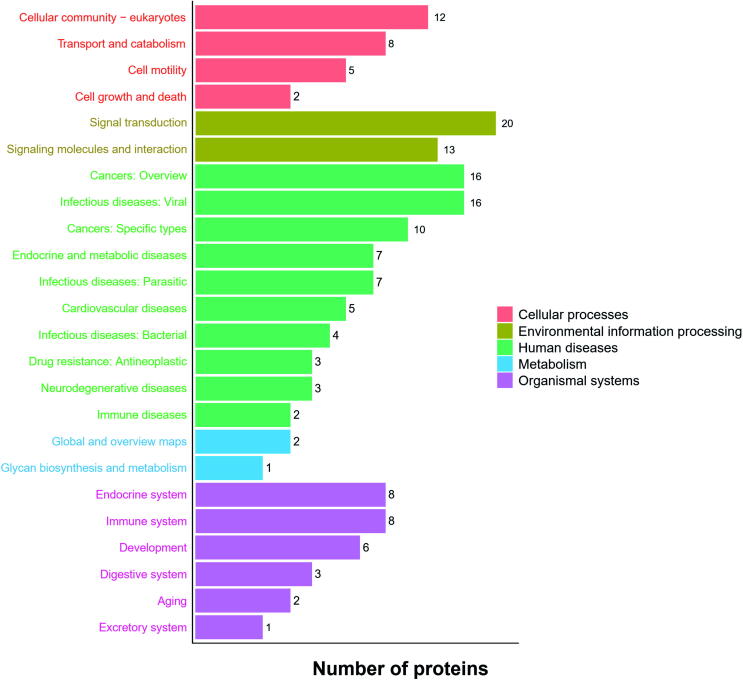
**KEGG analysis results of the identified bisecting GlcNAc containing glycoproteins**. KEGG, Kyoto Encyclopedia of Genes and Genomes.

To determine the potential functional roles of these glycoproteins with bisecting GlcNAc modification, Gene Ontology (GO) and Kyoto Encyclopedia of Genes and Genomes (KEGG) pathway analyses were performed. GO analysis of protein functions (Figure S4) indicated that these proteins might perform multiple functions, with 7 proteins (UniProt: P02786, Q8NES3, P30530, P04216, P05556, Q07954, and O00300) potentially involved in immune system processes. The analysis of KEGG pathways (Figure S5) indicated that the pathways involving the 41 glycoproteins can be classified into 5 categories: cellular processes, environmental information processing, human diseases, metabolism, and organismal systems. In the human disease category, 2 glycoproteins (UniProt: P0DOX2 and P10321) were shown to be related to immune diseases, and in the organismal system category, 8 of the glycoproteins (UniProt: O75339, P02786, P04216, P05556, P08473, P0DOX2, P10321, and Q7Z7G0) could play important roles in the immune system. Glycoprotein human leukocyte antigen (HLA) class I histocompatibility antigen C alpha chain (UniProt: P10321) requires further investigation as it appears in both categories.

## Conclusion

An integration strategy based on mass spectrometers was employed to determine whether bisected glycans of glycoproteins were present on the amniotic membrane. The MALDI–TOF MS spectrum provided an overview of the N-glycan profile on the amniotic membrane, and N-glycan at *m/z* 2489.4 was identified as a potential core-fucosylated biantennary bisected structure. Electrospray ionization (ESI) MS^n^ analyses further provided solid evidence for the identification of the bisecting GlcNAc characteristic ion at *m/z* 444.18, derived from the MS7 spectrum of the N-glycan at *m/z* 2489.4. Moreover, the glycopeptide bisected characteristic ions [Peptide + HexNAc_3_Hex] and [Peptide + FucHexNAc_3_Hex] upon MS2 signals assisted in the search for bisected glycans in large proteomics. Based on the overall MS evidence, we concluded that glycoproteins with bisected glycans are generally present on the human amniotic membrane.

Our glycomic and glycoproteomic analyses of the human amniotic membrane revealed, for the first time, that the N-glycan structure present on human gametes and trophoblasts is also expressed on the amniotic membrane, which provides additional evidence for the hu-FEDS hypothesis.

## Materials and methods

### Reagents and equipment

Chemical reagents were purchased from Sigma-Aldrich (St. Louis, MO) unless otherwise specified. Sequencing grade trypsin (V5111) was purchased from Promega (Madison, WI). Peptide N-glycosidase F (PNGase F; Catalog No. P0705L) was purchased from New England Biolabs (Beijing, China). Methanol (Catalog No. A412-4), acetonitrile (Catalog No. A996-4), and 2-propanol (Catalog No. A464-4) were obtained from ThermoFisher Scientific (Fair Lawn, NJ). Sep-Pak C18 cartridges (Catalog No. WAT054955) were purchased from Waters (Milford, MA). The mass spectrometers employed include a Bruker UltrafleXtreme MALDI–TOF/TOF mass spectrometer (Bremen, Germany) and a Thermo Scientific Orbitrap Fusion Lumos Tribrid mass spectrometer (San Jose, CA).

### Extraction of the human amniotic membrane proteins

Human amniotic membrane was isolated from the placenta [27]. Protein extraction from the amniotic membrane was performed as previously reported [32], [50]. The human amniotic placenta was lysed using a tissue lyser (JXESPRP, Jingxin Co., Shanghai, China) after washing with cold PBS. The lysates were then subjected to ultrasonication in 0.25 M sucrose containing the 1× protease inhibitor cocktail. The insoluble tissue fragments were removed by centrifugation of the lysis mixture at 260 *g* for 10 min at 4 °C. To further enrich membrane proteins, we performed ultracentrifugation, in which the membrane fraction was collected at 100,000 *g* for 1 h three times after washing with cold PBS. Proteins were extracted from the membrane fraction in 8 M urea and 20 mM Tris-HCl at pH 8.0. The extracted proteins were digested with Promega trypsin and desalted using Sep-Pak C18 cartridges. The protein concentrations of the homogenized amniotic membranes were determined by Bradford assay, and the rationality of the protein concentrations was verified using SDS–PAGE.

### Processing of the human amniotic membrane to acquire N-glycans

The membrane was subjected to standard protocols [51], [52]. Briefly, the membrane was suspended in lysis buffer before homogenization, and sonication was performed. The homogenates were reduced, carboxymethylated, and then dialyzed against 50 mM ammonia bicarbonate buffer at pH 7.5. Thereafter, the sample was lyophilized and then treated with trypsin. The treated sample was purified using a Sep-Pak C18 cartridge prior to the release of N-glycans by PNGase F digestion. The released N-glycans were permethylated and then purified using a Sep-Pak C18 cartridge prior to MS analysis.

### MS^n^ (*n* = 1–8) analyses for glycomics

The MS^n^ (*n* = 1–8) strategy to clarify the bisected N-glycans was divided into two steps. In the first step, MS data were obtained using a Bruker UltrafleXtreme MALDI–TOF/TOF mass spectrometer. Purified permethylated glycans were dissolved in 20 μl methanol, and 1 μl of the sample was mixed with 1 μl of matrix, 20 mg/ml dihydroxybenzoic acid in 70% (v/v) aqueous methanol, and loaded onto a metal target plate. The instrument was run in reflectron positive ion mode. In the second step, the MS (2–8) data were acquired using a Thermo Scientific Orbitrap Fusion Lumos Tribrid mass spectrometer. The permethylated glycans were dissolved in 1 mM NaOH in 50% methanol, and the solution was delivered to a mass spectrometer in direct infusion injection mode.

### High performance liquid chromatography separation for glycoproteome

The peptides were desalted and dissolved in high concentration organic solutions with shaking for 15 min at room temperature, and separated by centrifugation for 10 min at 16,000 *g*. Further high performance liquid chromatography separation details are presented in our previously published paper [32]. Briefly, both the supernatant and pellet peptides were separated on a self-packed capillary column N (180 μm × 4 cm, 3 μm, 120 Å) packed with Xtimate C18 (Welch, TX) at a flow rate of 8 μl/min on a Shimadzu high performance liquid chromatography system. The mobile phases consisted of A (deionized water) and B (50% acetonitrile, pH 9.0) with the following 70-min elution gradient: 0 min, 10% B; 5 min, 10% B; 60 min, 40% B; 62 min, 90% B; 64 min, 90% B; 65 min, 10% B; and 70 min, 10% B. The peptides were then collected and freeze-dried.

### Liquid chromatography–mass spectrometry

Each fraction was repeatedly injected to achieve a more confident identification. Peptide identification was conducted using a liquid chromatography–mass spectrometry system, in which a reversed-phase (RP) C18 column mounted on a Thermo Dionex UltiMate 3000 was coupled with a Thermo Scientific Orbitrap Fusion Lumos Tribrid mass spectrometer. The fractionated peptides were loaded onto an RP C18 column and eluted with a gradient of 5%–25% buffer B (98% acetonitrile, 2% H_2_O, and 1‰ formic acid) for 95 min, 25%–30% buffer B for 10 min, and 30%–80% buffer B for 5 min. The eluted peptides were then subjected to MS. The MS parameters were set at 120,000 for MS1 resolution and 30,000 for MS2 resolution with 30% normalized collision energy.

### Data analyses

The glycomic data were analyzed using flexAnalysis, Xcalibur, and GlycoWorkbench to obtain glycan structures. To verify the structure, the MS^n^ (*n* = 2–8) spectra related to bisecting GlcNAc-containing glycans were manually checked according to the approach in Figure 2. Notably, the peaks of precursor ions and corresponding fragment ions should be dominant in each MS^n^ spectrum, and the fragment ion mass difference between the observed *m/z* value and theoretical value should be different within the instrument resolution setting. Proteomic data were searched by Proteome Discoverer against the Swiss-Prot human database (version 2018-12-05). The false discovery rate was less than 1% at both the peptide-spectrum matches and protein levels during the search and was automatically calculated using the software [53], [54]. The glycoproteomic data were processed using a combination of Proteome Discoverer and Xcalibur to obtain the glycosylation sites and corresponding glycan components. The glycans and glycopeptides were further confirmed by manual examination of the corresponding MS2 spectra by checking for oxonium ions, monosaccharides, oligosaccharide neutral loss patterns, and bisected characteristic ions [Peptide + HexNAc_3_Hex] and [Peptide + FucHexNAc_3_Hex]. The raw proteomic data used in this study were obtained from our previous publication in 2018 [41] and are available in the public database (ProteomeXchange: PXD010630).

### Bioinformatic analysis of the glycoproteins containing bisecting GlcNAc

GO annotation was performed using the NCBI non-redundant database and Blast2GO software. Proteins were categorized into molecular function, cellular component, and biological process according to the GO terms [55], [56]. KEGG (version 89.1) and Blast2KO software were used to annotate the pathways [57], [58]. The *P* < 0.05 was considered significant.

## Ethical statement

The human placenta used in this study was approved by the BGI Institute of Review Board of Bioethics and Biosafety (Approval No. BGI-IRB 18084). The written informed consent was obtained from the participating subject.

## Data availability

The raw glycomic data (MS1–MS8) generated in this study have been uploaded to China National GeneBank DataBase (CNSA: CNP0001701), which are publicly accessible at https://db.cngb.org/search/project/CNP0001701/.

## CRediT author statement

**Qiushi Chen:** Conceptualization, Methodology, Software, Investigation, Writing - original draft, Visualization. **Yuanliang Zhang:** Methodology, Software, Formal analysis, Investigation, Visualization. **Keren Zhang:** Formal analysis, Investigation. **Jie Liu:** Software, Formal analysis, Visualization. **Huozhen Pan:** Visualization, Investigation. **Xinran Wang:** Software, Visualization. **Siqi Li:** Investigation. **Dandan Hu:** Investigation. **Zhilong Lin:** Formal analysis, Investigation. **Yun Zhao:** Investigation. **Guixue Hou:** Validation, Investigation. **Feng Guan:** Validation. **Hong Li:** Validation. **Siqi Liu:** Resources, Writing - review & editing, Visualization, Supervision, Project administration. **Yan Ren:** Conceptualization, Resources, Writing - review & editing, Visualization, Supervision, Project administration, Funding acquisition. All authors have read and approved the final manuscript.

## Competing interests

The authors have declared no competing interests.

## Supplementary material

Supplementary figure S2 **MALDI–TOF MS spectra annotation**. Annotated MALDI–TOF MS spectra of permethylated N-glycans from human **A**. colon, **B**. heart, **C**. kidney, and **D**. amniotic membrane. All panels show that the glycans in the mass range from m/z 1500 to 3250. All ions are [M + Na]^+^. Peaks are labeled with their m/z values, and putative structures are described based on the molecular weight and N-glycan biosynthetic pathway. The N-glycan profiles of the human colon, heart, and kidney were obtained from the Consortium for Functional Glycomics. The extensive data sets that have resulted from the use of Consortium for Functional Glycomics resources are publicly available through the website and include results from glycan array screening, glycogene microarray screening, mouse phenotyping, and glycan profiling experiments.

Supplementary figure S3 **ESI MSn spectra annotation**. **A**. Annotated ESI FTMS MS2 spectrum of permethylated N-glycan at m/z 2489.25 from the human amniotic membrane. **B**. Annotated ESI ITMS MS3 spectrum of permethylated N-glycan ion at m/z 2489.25 from the human amniotic membrane. **C**. Annotated ESI ITMS MS4 spectrum of permethylated N-glycan ion at m/z 2489.25 from the human amniotic membrane. **D**. Annotated ESI ITMS MS5 spectrum of permethylated N-glycan ion at m/z 2489.25 from the human amniotic membrane. **E**. Annotated ESI ITMS MS6 spectrum of permethylated N-glycan ion at m/z 2489.25 from the human amniotic membrane. **F**. Annotated ESI ITMS MS8 spectrum of permethylated N-glycan ion at m/z 2489.25 from the human amniotic membrane. Assignments of the possible fragment ions are indicated on the cartoons and on the spectrum. Ions with different charges are labeled using different colors. The number indicated above the peak is the m/z value of the fragment ion (resulting ion) that has been detected by the mass spectrometer. Data were acquired in the form of [M + Na]^+^ or [M + 2Na]2^+^. FTMS, Fourier transform mass spectrometry.
