# Supplementary figures and images for: Profiling the Bisecting N-acetylglucosamine Modification in Amniotic Membrane via Mass Spectrometry
Source: Genomics Proteomics Bioinformatics. 2022 Feb 3;20(4):648–56. doi: 10.1016/j.gpb.2021.09.010 (PMC9880894; doi:10.1016/j.gpb.2021.09.010)

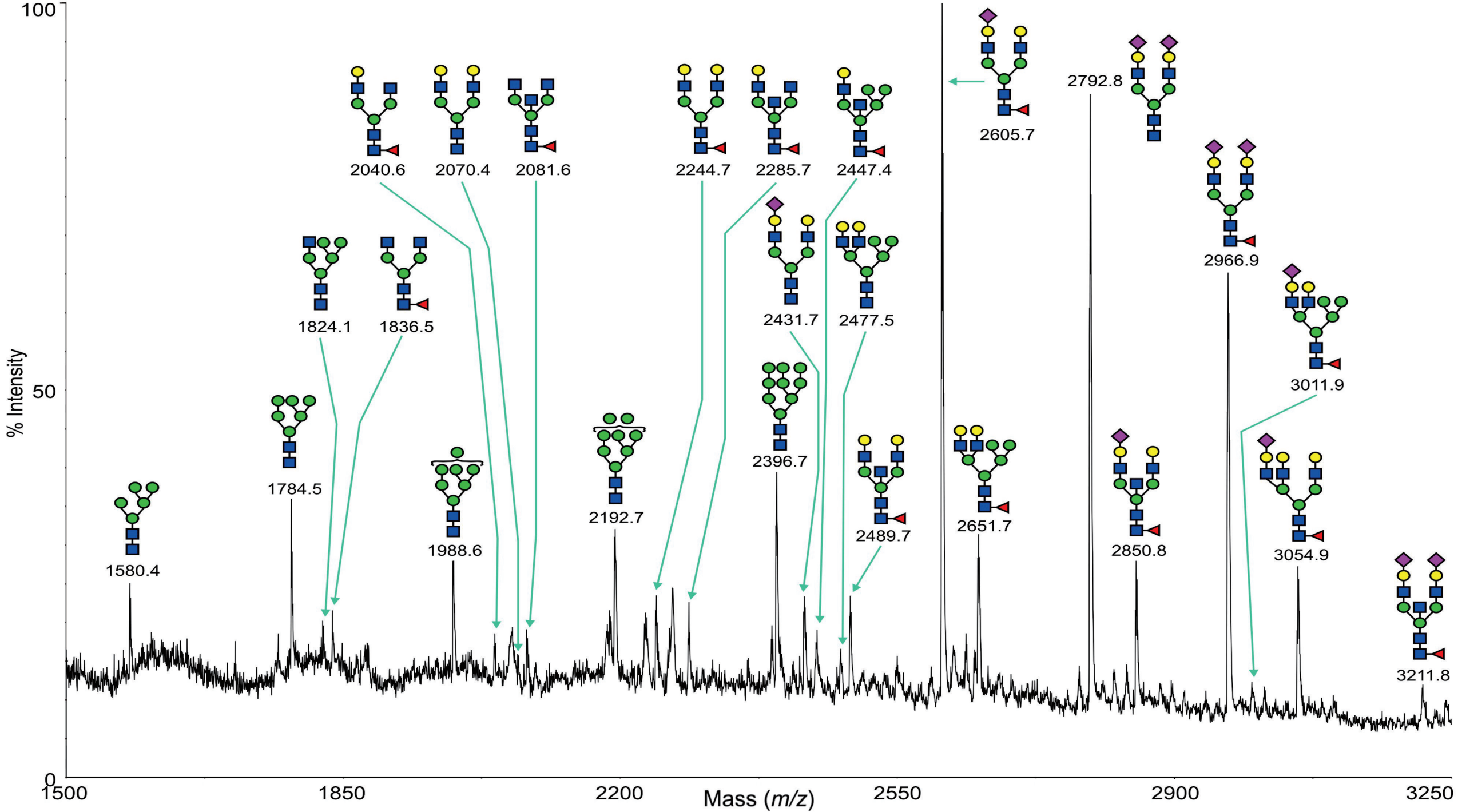

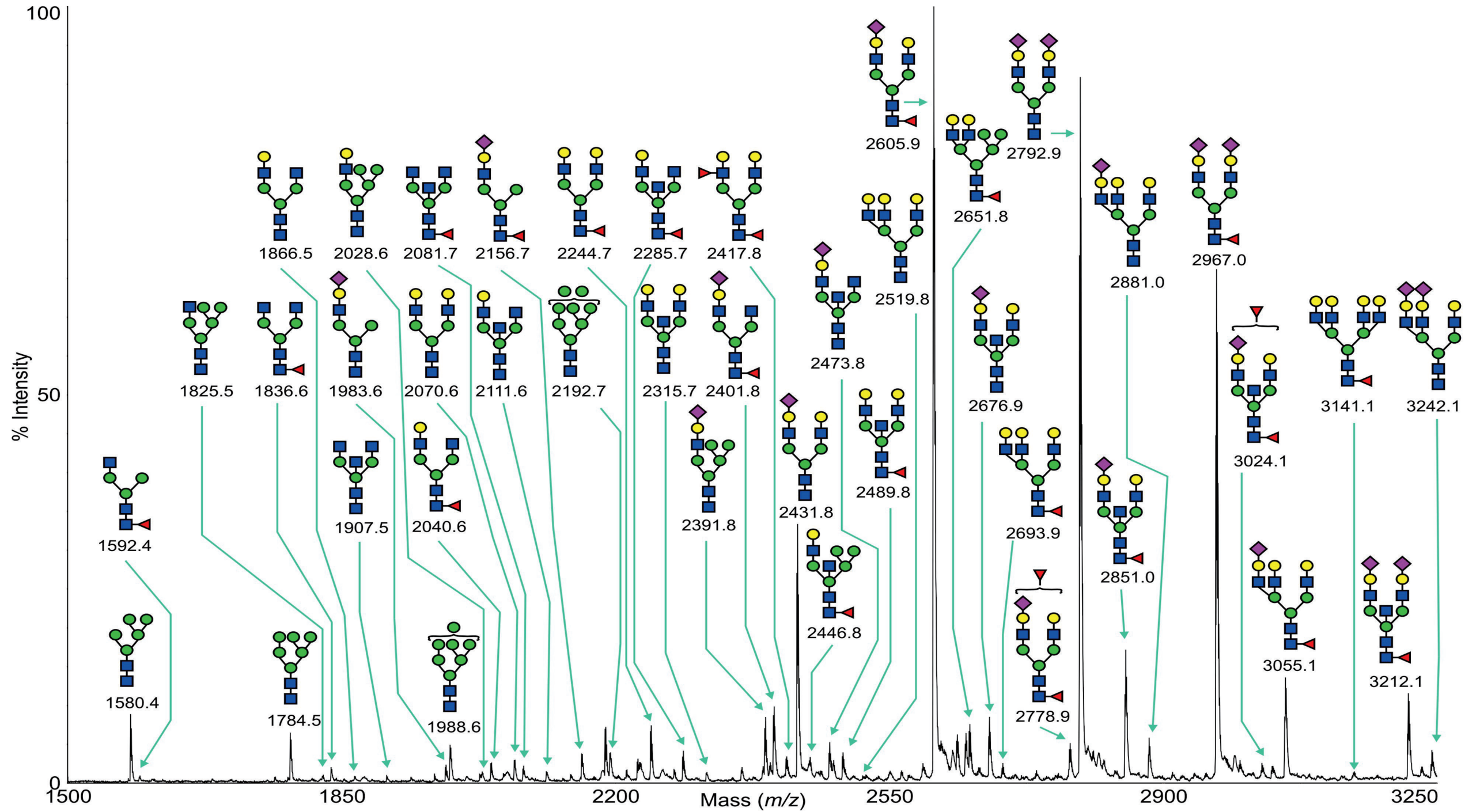

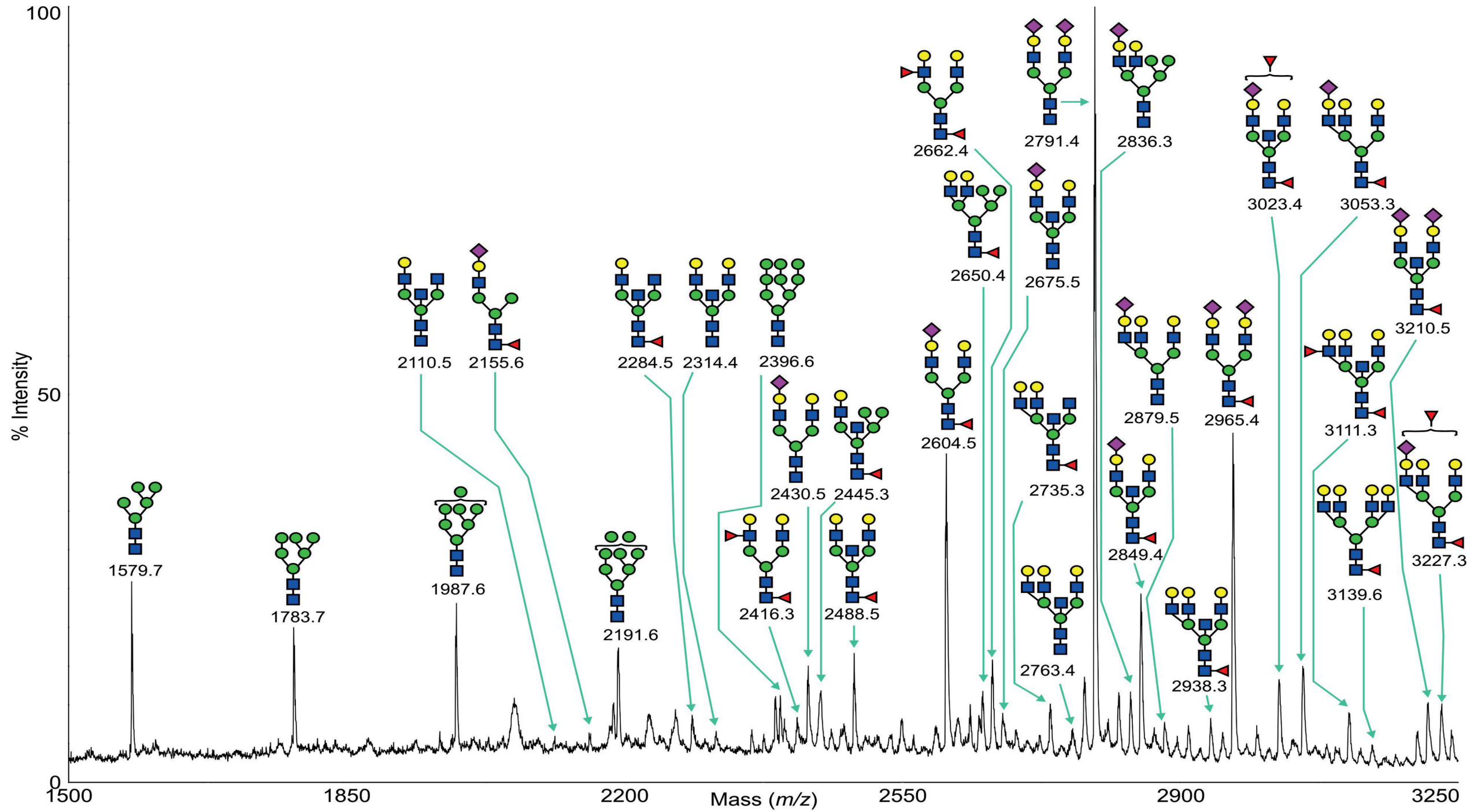

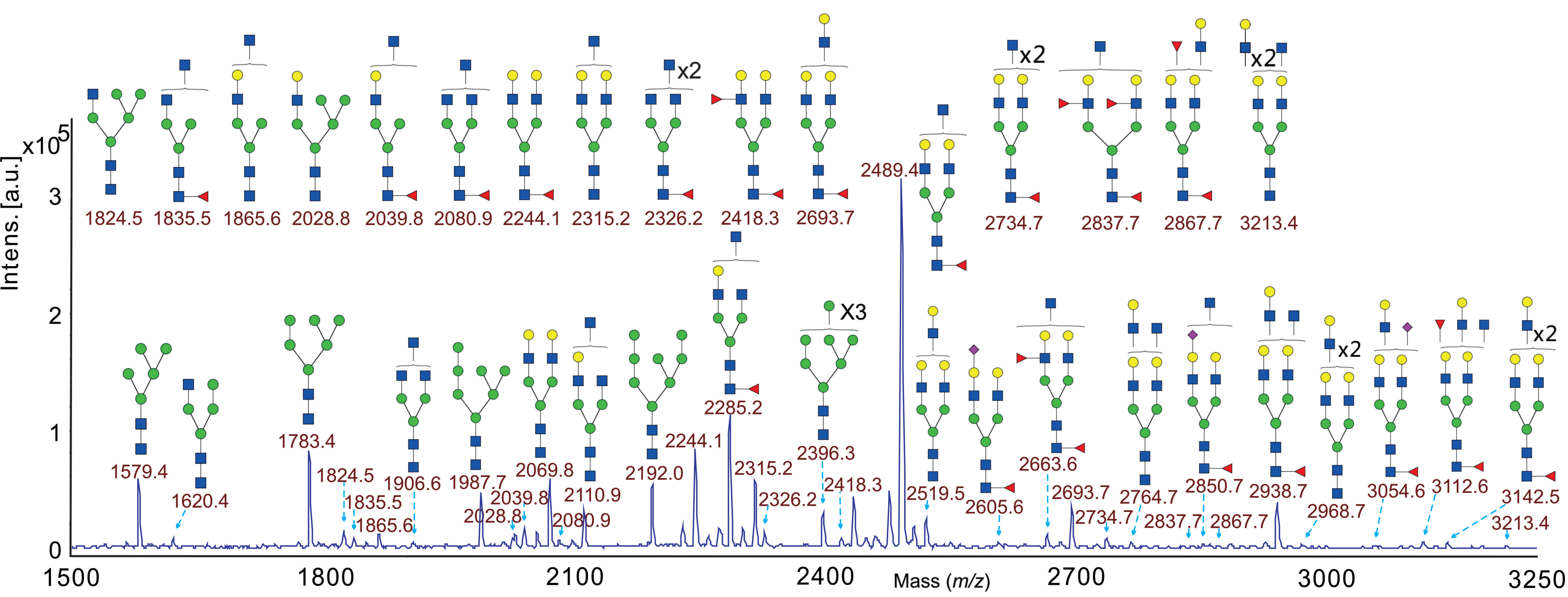

Supplement: Supplementary figure S2 — MALDI–TOF MS spectra annotation. Annotated MALDI–TOF MS spectra of permethylated N-glycans from human A. colon, B. heart, C. kidney, and D. amniotic membrane. All panels show that the glycans in the mass range from m/z 1500 to 3250. All ions are [M + Na]+. Peaks are labeled with their m/z values, and putative structures are described based on the molecular weight and N-glycan biosynthetic pathway. The N-glycan profiles of the human colon, heart, and kidney were obtained from the Consortium for Functional Glycomics. The extensive data sets that have resulted from the use of Consortium for Functional Glycomics resources are publicly available through the website and include results from glycan array screening, glycogene microarray screening, mouse phenotyping, and glycan profiling experiments. [file mmc1.pdf]

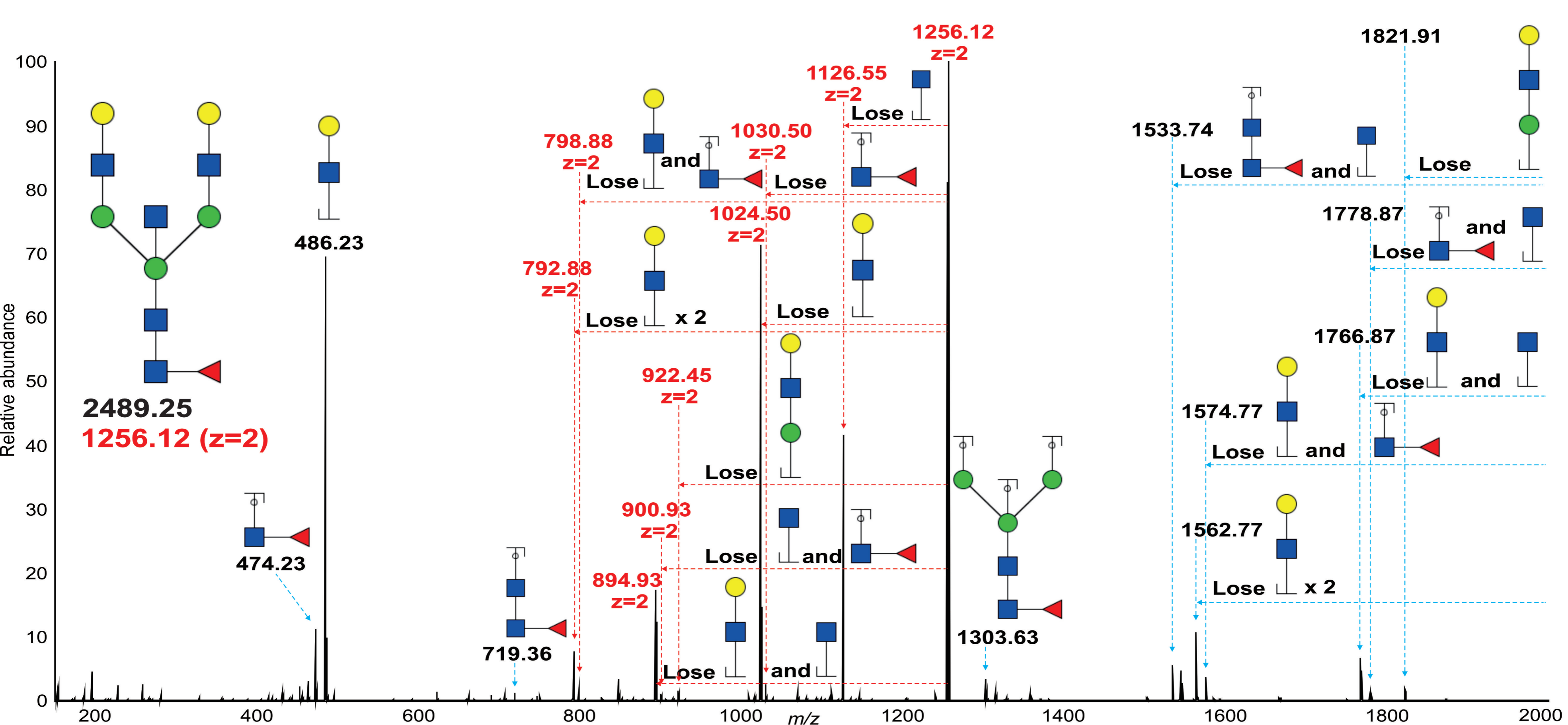

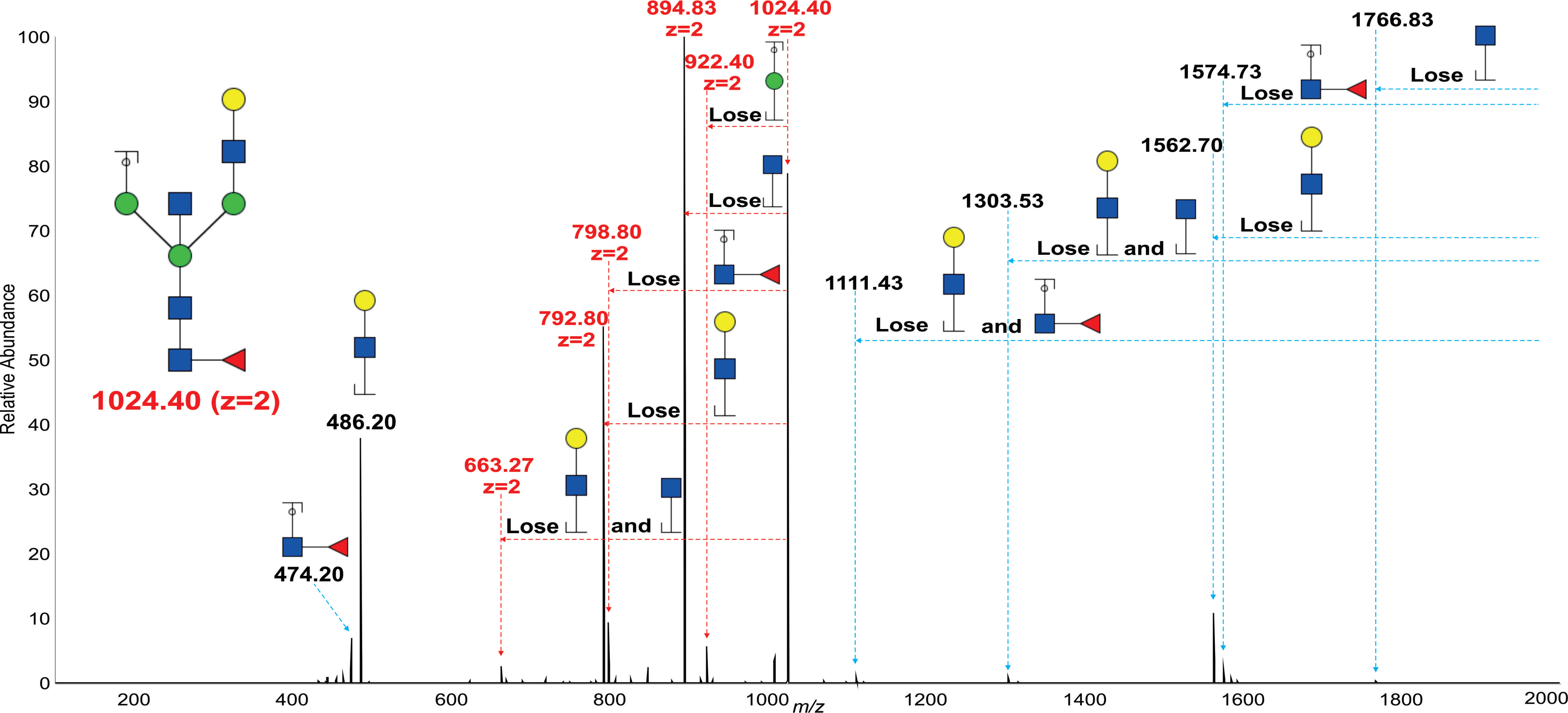

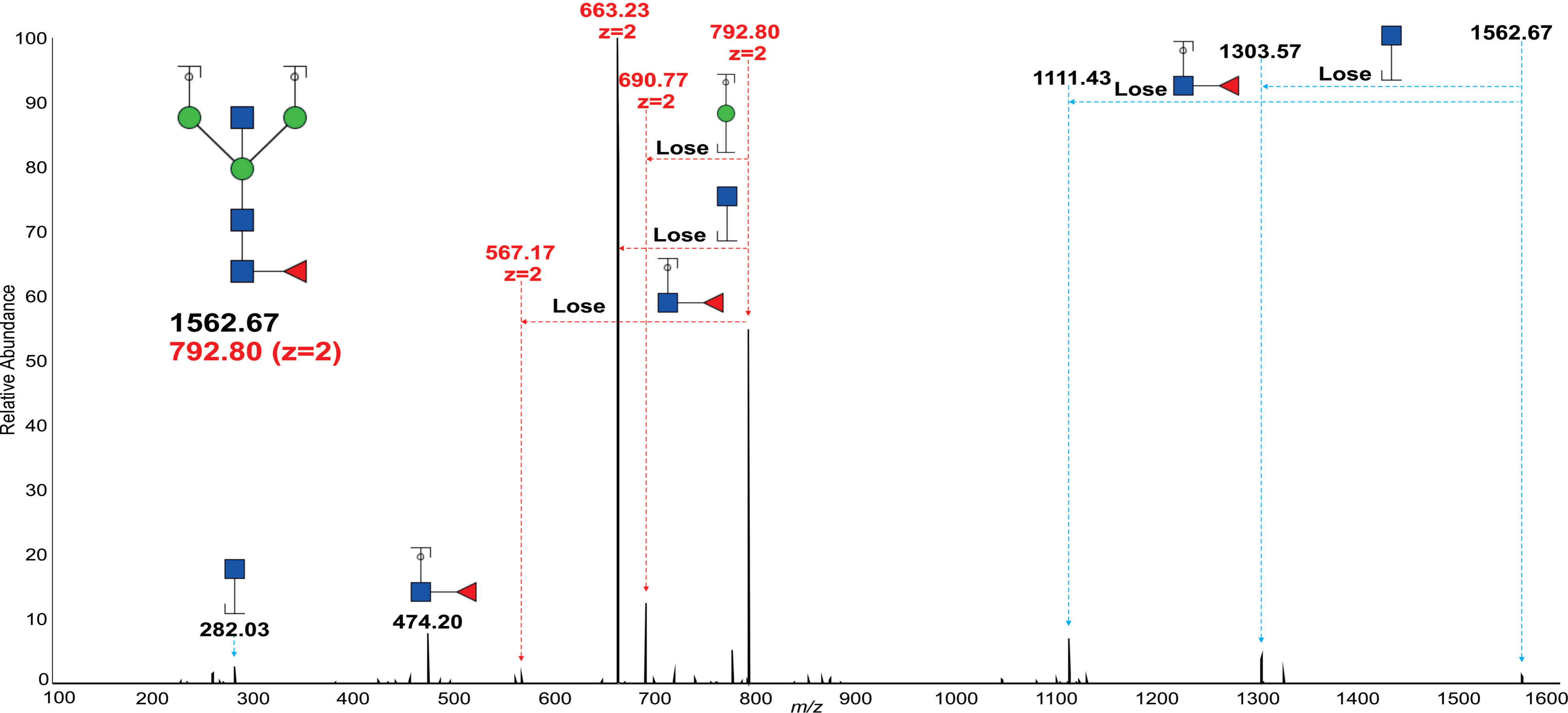

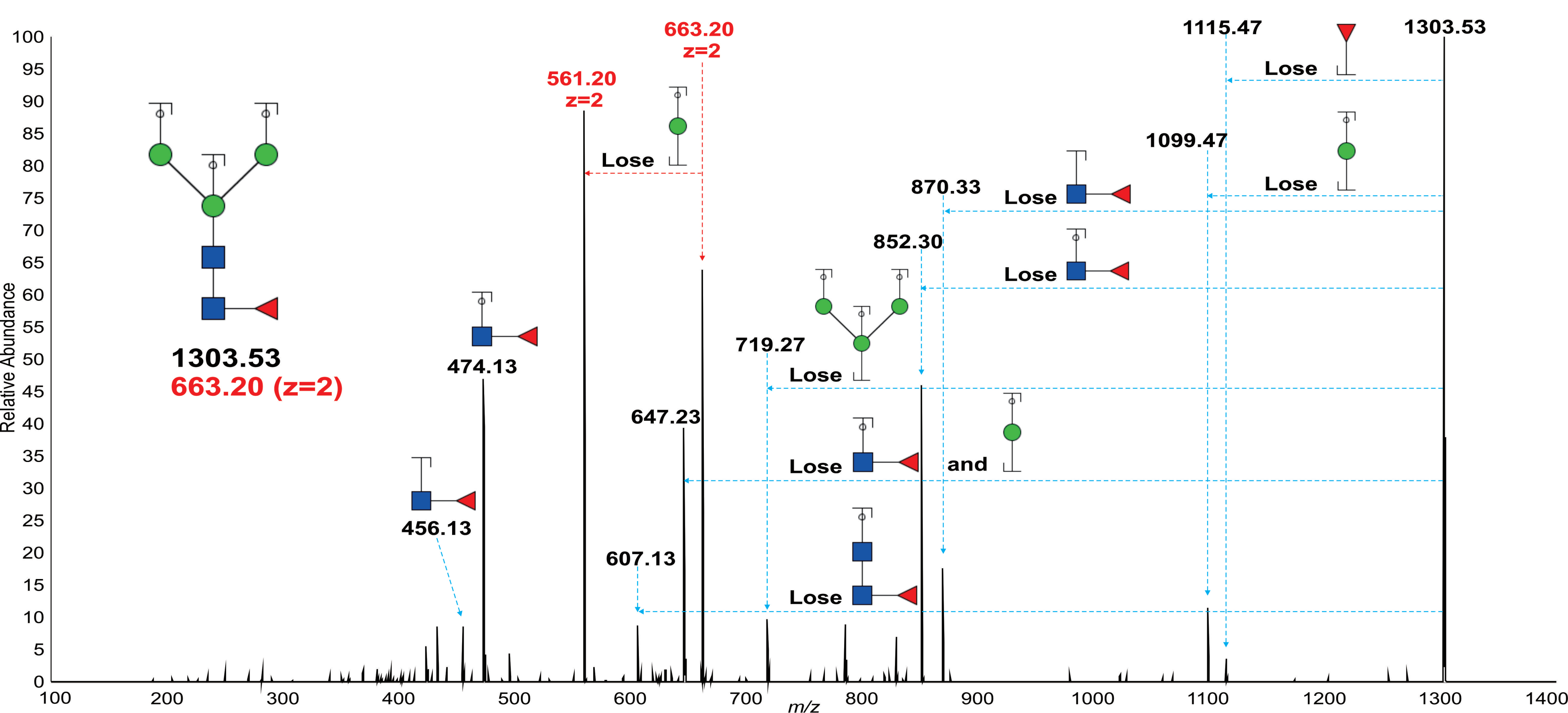

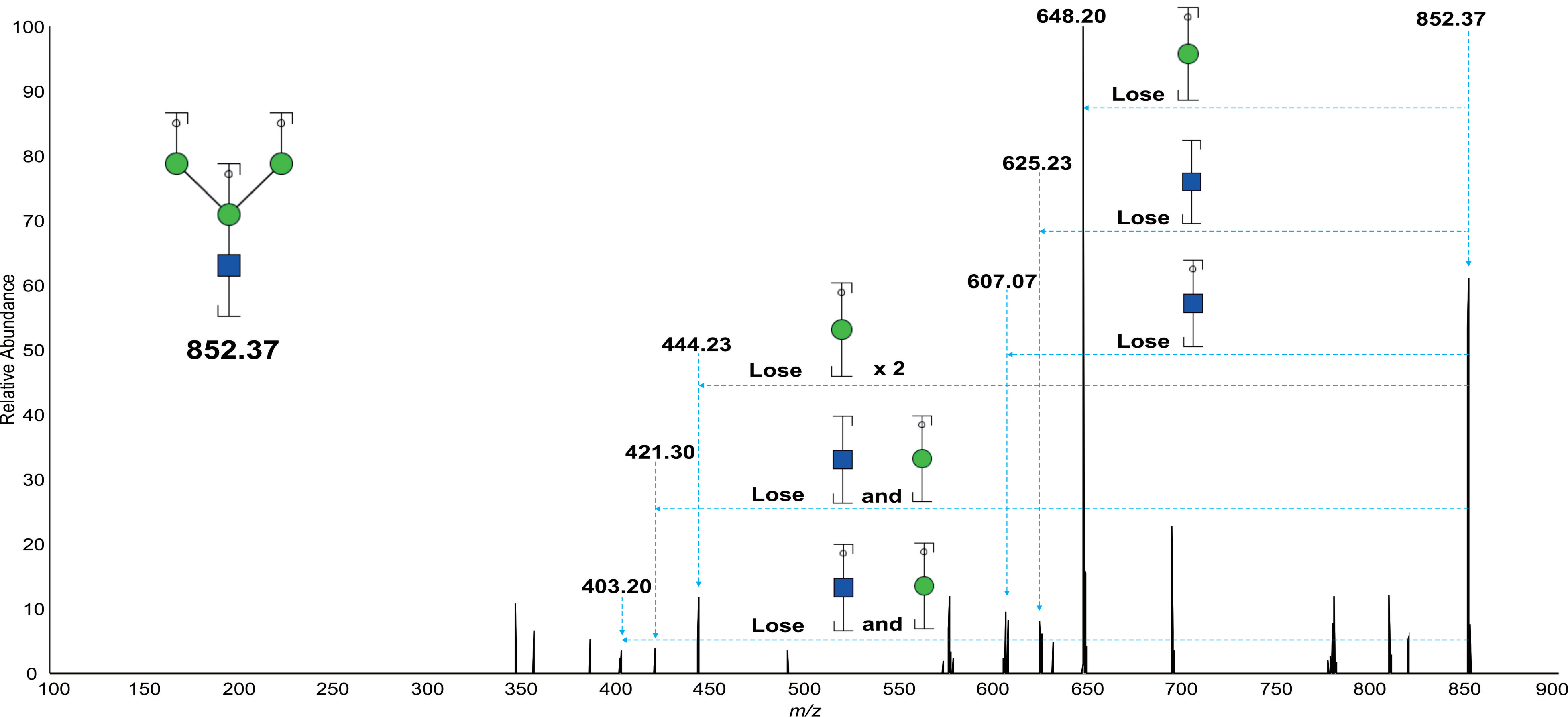

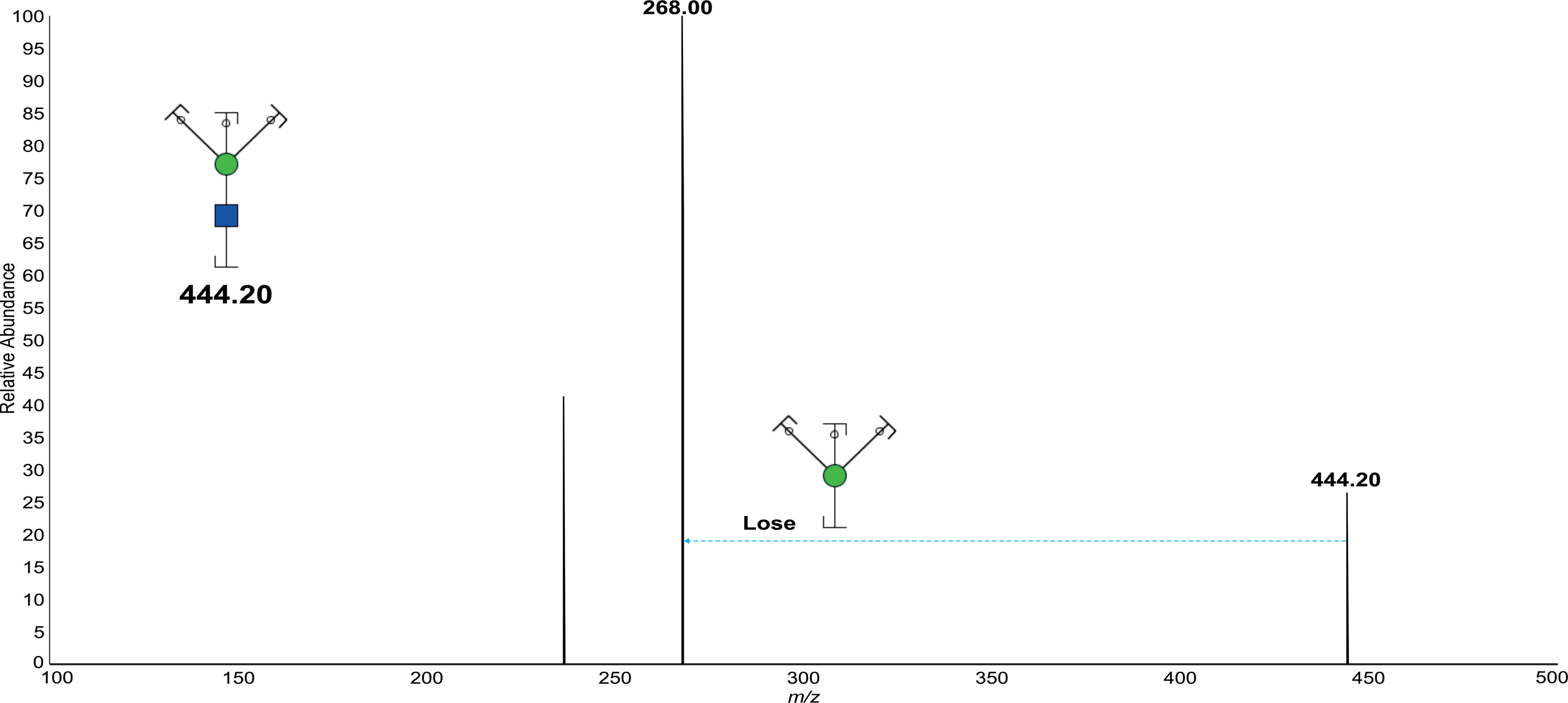

Supplement: Supplementary figure S3 — ESI MSn spectra annotation. A. Annotated ESI FTMS MS2 spectrum of permethylated N-glycan at m/z 2489.25 from the human amniotic membrane. B. Annotated ESI ITMS MS3 spectrum of permethylated N-glycan ion at m/z 2489.25 from the human amniotic membrane. C. Annotated ESI ITMS MS4 spectrum of permethylated N-glycan ion at m/z 2489.25 from the human amniotic membrane. D. Annotated ESI ITMS MS5 spectrum of permethylated N-glycan ion at m/z 2489.25 from the human amniotic membrane. E. Annotated ESI ITMS MS6 spectrum of permethylated N-glycan ion at m/z 2489.25 from the human amniotic membrane. F. Annotated ESI ITMS MS8 spectrum of permethylated N-glycan ion at m/z 2489.25 from the human amniotic membrane. Assignments of the possible fragment ions are indicated on the cartoons and on the spectrum. Ions with different charges are labeled using different colors. The number indicated above the peak is the m/z value of the fragment ion (resulting ion) that has been detected by the mass spectrometer. Data were acquired in the form of [M + Na]+ or [M + 2Na]2+. FTMS, Fourier transform mass spectrometry. [file mmc2.pdf]
