# Supplementary material for: Profiling the Bisecting N-acetylglucosamine Modification in Amniotic Membrane via Mass Spectrometry
Source: Genomics Proteomics Bioinformatics. 2022 Feb 3;20(4):648–56. doi: 10.1016/j.gpb.2021.09.010 (PMC9880894; doi:10.1016/j.gpb.2021.09.010)
Supplement: Supplementary Table S1 [file mmc3.docx]

**Table S1 Detailed information of all identified glycoproteins**

| Accession No. | Protein No. | Glycosylated peptide sequence | Cellular location |
| --- | --- | --- | --- |
| O00300 | Tumor necrosis factor receptor superfamily member 11B | HT**N**C(carbamidomethyl)SVFGLLLTQK | Extracellular matrix |
| O15230 | Laminin subunit alpha-5 | GVH**N**ASLALSASIGR; D**N**ATLQATLHAAR | Extracellular matrix |
| O75339 | Cartilage intermediate layer protein 1 | LPHDC(carbamidomethyl)FQ**N**ATNSFYYDVGR | Extracellular matrix |
| P02751 | Fibronectin | RHEEGHML**N**C(carbamidomethyl)TC(carbamidomethyl)FGQGR | Extracellular matrix |
| P02786 | Transferrin receptor protein 1 | KDFEDLYTPV**N**GSIVIVR | Cell membrane |
| P04216 | Thy-1 membrane glycoprotein | HE**N**TSSSPIQYEFSLTR | Cell membrane |
| P04628 | Proto-oncogene Wnt-1 | C(carbamidomethyl)**N**C(carbamidomethyl)TFHWC(carbamidomethyl)C(carbamidomethyl)HVSC(carbamidomethyl)R | Extracellular matrix |
| P05556 | Integrin beta-1 | LRNPC(carbamidomethyl)TSEQ**N**C(carbamidomethyl)TSPFSYK | Cell membrane |
| P06213 | Insulin receptor | C(carbamidomethyl)V**N**FSFC(carbamidomethyl)QDLHHK | Cell membrane |
| P06756 | Integrin alpha-V | ISSLQTTEK**N**DTVAGQGER | Cell membrane |
| P07585 | Decorin | LGLSFNSISAVD**N**GSLANTPHLR; YIQVVYLHNN**N**ISVVGSSDFC(carbamidomethyl)PPGHNTK | Extracellular matrix |
| P08473 | Neprilysin | EIA**N**ATAKPEDR | Cell membrane |
| P0DOX2 | Immunoglobulin alpha-2 heavy chain | HYT**N**SSQDVTVPC(carbamidomethyl)R | Extracellular matrix |
| P10321 | HLA class I histocompatibility antigen, C alpha chain | GYY**N**Q(deamidated)SEDGSHTLQR; | Cell membrane |
| P12111 | Collagen alpha-3(VI) chain | Q(Gln->pyro-Glu)LINALQI**N**NTAVGHALVLPAGR | Extracellular matrix |
| P13473 | Lysosome-associated membrane glycoprotein 2 | VASVININP**N**TTHSTGSC(carbamidomethyl)R | Cell membrane |
| P13688 | Carcinoembryonic antigen-related cell adhesion molecule 1 | **N**QSLPSSER | Cell membrane |
| P14384 | Carboxypeptidase M | TVAQ**N**YSSVTHLHSIGK | Cell membrane |
| P15328 | Folate receptor alpha | NAC(carbamidomethyl)C(carbamidomethyl)ST**N**TSQEAHKDVSYLYR | Cell membrane |
| P16144 | Integrin beta-4 | HNIIPIFAVT**N**YSYSYYEK | Cell membrane |
| P20774 | Mimecan | A**N**DTSYIR | Extracellular matrix |
| P30530 | Tyrosine-protein kinase receptor UFO | **N**GSQAFVHWQEPR | Cell membrane |
| P35556 | Fibrillin-2 | C(carbamidomethyl)NSGFALDMEER**N**C(carbamidomethyl)TDIDEC(carbamidomethyl)R | Extracellular matrix |
| P41221 | Protein Wnt-5a | **N**ESTGSLGTQGR | Extracellular matrix |
| P42892 | Endothelin-converting enzyme 1 | HLLE**N**STASVSEAER | Cell membrane |
| P43121 | Cell surface glycoprotein MUC18 | C(carbamidomethyl)GLSQSQG**N**LSHVDWFSVHK | Cell membrane |
| P51884 | Lumican | KLHINHN**N**LTESVGPLPK | Extracellular matrix |
| P51888 | Prolargin | NSF**N**ISNLLVLHLSHNR | Extracellular matrix |
| P55268 | Laminin subunit beta-2 | RA**N**TSALAVPSPVSNSASAR | Extracellular matrix |
| P98095 | Fibulin-2 | SC(carbamidomethyl)KDVDEC(carbamidomethyl)ALGTH**N**C(carbamidomethyl)SEAETC(carbamidomethyl)HNIQGSFR | Extracellular matrix |
| P98160 | Basement membrane-specific heparan sulfate proteoglycan core protein | NQELEDNVHISP**N**GSIITIVGTRPSNHGTYR | Extracellular matrix |
| Q01638 | Interleukin-1 receptor-like 1 | FIHNENGA**N**YSVTATR | Cell membrane |
| Q07954 | Prolow-density lipoprotein receptor-related protein 1 | MHL**N**GSNVQVLHR | Cell membrane |
| Q14112 | Nidogen-2 | IHQ**N**ITYQVC(carbamidomethyl)R | Extracellular matrix |
| Q14766 | Latent-transforming growth factor beta-binding protein 1 | G**N**TTTLISEN(deamidated)GHAADTLTATNFR | Extracellular matrix |
| Q16787 | Laminin subunit alpha-3 | **N**ASGDELVR | Extracellular matrix |
| Q6ZMJ2 | Scavenger receptor class A member 5 | GL**N**HSLSYDVALHR | Cell membrane |
| Q7Z7G0 | Target of Nesh-SH3 | TQLAK**N**ETLALPAESK | Extracellular matrix |
| Q8NES3 | Beta-1,3-N-acetylglucosaminyltransferase lunatic fringe | HTGNVVIT**N**C(carbamidomethyl)SAAHSR | Golgi apparatus membrane |
| Q9BXX0 | EMILIN-2 | DAYVEAVLSVS**N**ASVAQLHTAGYR | Extracellular matrix |
| Q9H1J7 | Protein Wnt-5b | **N**ESTGSLGTQGR | Extracellular matrix |

*Note*: Detailed information includes accession number, protein name, one glycosylated peptide sequence possessing bisecting GlcNAc, and the cellular location of each glycoprotein. The N-glycosylation site in each peptide is marked using red, and the cellular location was obtained via searches in the UniProt library. GlcNAc, N-acetylglucosamine.
